# Neurocognitive, psychiatric, and substance use characteristics in a diverse sample of persons with OUD who are starting methadone or buprenorphine/naloxone in opioid treatment programs

**DOI:** 10.1186/s13722-021-00272-4

**Published:** 2021-10-24

**Authors:** Travis M. Scott, Julia Arnsten, James Patrick Olsen, Franchesca Arias, Chinazo O. Cunningham, Monica Rivera Mindt

**Affiliations:** 1 VA Palo Alto Health Care System Sierra Pacific Mental Illness Research Education Clinical Center, 3801 Miranda Ave, Palo Alto, CA 94304 USA; 2 Department of Psychiatry and Behavioral Sciences, Stanford School of Medicine, Stanford, CA USA; 3 Department of Medicine, Albert Einstein College of Medicine and Montefiore Medical Center, Bronx, NY USA; 4 Department of Neurology, North Shore University Hospital, Manhasset, NY USA; 5 The Aging Brain Center, Hebrew SeniorLife, Boston, MA USA; 6 Department of Cognitive Neurology, Beth Israel Deaconess Medical Center, Harvard Medical School, Boston, MA USA; 7 Department of Psychology and Latin America and Latino Studies Institute, Fordham University, New York, NY USA; 8 Department of Neurology, Icahn School of Medicine at Mount Sinai, New York, NY USA

**Keywords:** Opioid use disorder, Methadone, Buprenorphine/naloxone, Learning and memory, Depression, Comorbid substance use

## Abstract

**Background:**

Medications for opioid use disorder such as opioid agonist treatment (OAT, including methadone, buprenorphine) are the gold standard intervention for opioid use disorder (OUD). Persons with OUD have high rates of neurocognitive impairment and psychiatric and substance use disorders, but few studies have examined these characteristics in diverse patients initiating OAT in opioid treatment programs (OTPs). Additionally, in these individuals, poor neurocognitive functioning and psychiatric/other substance use disorders are associated with poor OUD treatment outcomes. Given rapid changes in the opioid epidemic, we sought to replicate findings from our pilot study by examining these characteristics in a large diverse sample of persons with OUD starting OTP-based OAT.

**Methods:**

Ninety-seven adults with OUD (*M* age = 42.2 years [*SD* = 10.3]; *M* education = 11.4 years [*SD* = 2.3]; 27% female; 22% non-Hispanic white) were enrolled in a randomized longitudinal trial evaluating methadone versus buprenorphine/naloxone on neurocognitive functioning. All participants completed a comprehensive neurocognitive, psychiatric, and substance use evaluation within one week of initiating OAT.

**Results:**

Most of the sample met criteria for learning (79%) or memory (69%) impairment. Half exhibited symptoms of current depression, and comorbid substance use was highly prevalent. Lifetime cannabis and cocaine use disorders were associated with better neurocognitive functioning, while depression was associated with worse neurocognitive functioning.

**Conclusions:**

Learning and memory impairment are highly prevalent in persons with OUD starting treatment with either methadone or buprenorphine/naloxone in OTPs. Depression and comorbid substance use are prevalent among these individuals, but neither impact learning or memory. However, depression is associated with neurocognitive impairment in other domains. These findings might allow clinicians to help persons with OUD starting OAT to develop compensatory strategies for learning and memory, while providing adjunctive treatment for depression.

*Trial Registration* NCT, NCT01733693. Registered November 4, 2012, https://clinicaltrials.gov/ct2/show/NCT01733693.

## Background

Medication treatment with opioid agonist treatment (OAT, including methadone, buprenorphine, or buprenorphine/naloxone) is the gold standard intervention for opioid use disorder (OUD; [1]). Methadone is most often provided in physician monitored, long-term outpatient opioid treatment programs (OTPs), and buprenorphine/naloxone is becoming increasingly common in the United States (U.S.) among individuals seeking treatment for OUD [2]. Reporting on important characteristics of persons with OUD initiating OAT in OTPs, such as neurocognitive abilities, psychiatric conditions, and comorbid substance use, is vital to understanding and improving treatment outcomes in these individuals.

Poor neurocognitive functioning is associated with poor substance use treatment outcomes, such as higher relapse and lower substance abstinence rates [3, 4]. Individual studies and systematic reviews indicate that chronic opioid use is associated with impaired learning, memory, attention/working memory, and executive functioning [5–11]. More recent studies have characterized cognitive impairments in persons with OUD already engaged in OAT with methadone [12] or buprenorphine [13] and have found similar domains of cognitive impairment (i.e., attention/working memory and executive functioning). While these studies demonstrate adverse neurocognitive effects of chronic opioid use and describe cognitive characteristics of those already engaged in opioid agonist treatment, only two studies to our knowledge have characterized the neurocognitive profiles of persons with OUD who are *starting* OAT [5, 14]. While these studies found impairments in several neurocognitive domains including learning, memory, executive functioning, and motor skills, both were limited by inadequate or outdated neurocognitive batteries and small sample sizes, suggesting that more research is needed to understand the neurocognitive characteristics of persons with OUD who initiate OAT in OTPs.

Psychiatric disorders (e.g., depression) and other, non-opioid substance use are associated with a host of factors that can complicate OAT (e.g., poor health, high rates of criminal activities; [15, 16]). Both depression [17] and additional substance use [18] are more prevalent in persons with an OUD than in the general population. While studies have examined the psychiatric and/or substance use characteristics of patients starting (or initiating) OAT [5, 19, 20], only one study to date has examined associations between psychiatric disorders, substance use, and neurocognitive function in persons with OUD who are initiating OAT. This study found high rates of lifetime major depressive disorder (31%), moderate to severe current depressive symptomatology (28%), and high rates (i.e., > 30%) of lifetime and current alcohol, cannabis, and cocaine use among OUD patients [5]. Arias and colleagues [5] also found that persons with OUD who had a lifetime history of alcohol dependence had worse global neurocognitive functioning than those without, and that persons with OUD who had a lifetime history of cocaine dependence had worse attention/working memory and motor functioning than those without, and concluded that there may be a synergistic effect of multiple substance use disorders contributing to neurocognitive impairment in persons with OUD.

The opioid epidemic has rapidly shifted in the past several years from being initially fueled by heroin and opioid analgesics to most recently synthetic opioids (e.g., fentanyl; [21]). As such, the objective of our study was to replicate, expand, and update the findings of our pilot study [5] by examining neurocognitive, psychiatric, and substance use characteristics of a larger, more diverse group of persons with OUD who were initiating OAT with either methadone or buprenorphine/naloxone. To achieve this objective, we analyzed baseline data from a larger randomized trial examining how methadone and buprenorphine/naloxone affect neurocognitive functioning. We expected high rates of neurocognitive impairment (i.e., in domains of learning, memory, attention/working memory, and executive functioning), depression, and comorbid substance use. We hypothesized that persons with OUD who had lifetime diagnoses of alcohol or cocaine use disorder would have worse neurocognitive functioning than those without either condition. Although Arias and colleagues [5] found that lifetime major depression was not associated with neurocognitive functioning in persons with OUD, based on previous literature [22] we also hypothesized that depressed persons with OUD would have worse neurocognitive functioning than non-depressed persons with OUD.

## Methods

### Participants

The sample included 97 English-speaking adults with opioid use disorder (OUD) starting opioid agonist treatment (OAT) with either methadone or buprenorphine/naloxone. Participants were recruited from and assessed in three clinics that together comprise the Einstein/Montefiore Division of Substance Abuse (DoSA) in the Bronx, New York. Recruitment was both active (e.g., approaching new patients at DoSA clinics) and passive (e.g., patients self-referred after seeing flyers posted in DoSA clinics, hearing about the study through word-of-mouth, or seeing a study advertisement in a local newspaper). Participants were enrolled in a randomized trial comparing neurocognitive outcomes among persons with OUD who were initiating methadone versus buprenorphine/naloxone; the analysis presented here is from the baseline evaluation.

Eligibility criteria for the randomized trial included: (1) diagnosis of an OUD without pharmacological treatment for OUD within the previous 90 days, (2) no current use of street methadone or buprenorphine/naloxone and no current prescription for either of these medications, (3) English-speaking, (4) between the ages of 18 and 68, (5) completed six or more years of education, and (6) able to provide informed consent (e.g., not acutely intoxicated at time of enrollment). We excluded persons with comorbid illness likely to impact neurocognitive functioning, including medical (i.e., liver disease, cardiovascular disease, oxygen-requiring lung disease, or end stage renal disease), neurological (i.e., history of head injury with loss of consciousness > 24 h, focal brain lesion, prior neurosurgery, non-alcohol related seizure disorder, or history of non-HIV CNS opportunistic infection), or psychiatric comorbidity other than major depression (i.e., schizophrenia or bipolar disorder). The trial was approved by the Institutional Review Boards of both Albert Einstein College of Medicine/Montefiore Medical Center and Fordham University.

### Measures

#### Neurocognitive functioning

Table 1 summarizes the comprehensive, standardized neurocognitive test battery participants completed, including tests in the following seven domains: executive functioning, learning, memory, attention/working memory, processing speed, motor abilities, and verbal fluency. Participants also completed the Wide Range Achievement Test-Reading subtest, 3rd edition (WRAT-3; [23]) as a measure of premorbid intellectual functioning.

**Table 1:** Neuropsychological battery and normative data by seven major neurocognitive domains

Neuropsychological Domain and Test	Normative Data Sources
Executive functioning	
Wisconsin Card Sorting Task-64 Item	[29]^a,b^
Trail Making Test (Part B)	[28]^a,b,c,d^
Learning	
Hopkins Verbal Learning Test—Revised	[26]^a^
Brief Visuospatial Memory Test—Revised	[25]^a^
Memory	
Hopkins Verbal Learning Test—Revised	[26]^a^
Brief Visuospatial Memory Test—Revised	[25]^a^
Attention/Working Memory	
WAIS-IV Letter Number Sequencing	[30]^a,b,c,d^
PASAT-50 Total Correct	[27]^a,b,d^
Speed of Information Processing	
WAIS-IV Coding	[30]^a,b,c,d^
WAIS-IV Symbol Search	[30]^a,b,c,d^ [28]^a,b,c,d^
Trail Making Test (Part A)	
Motor Skills	
Grooved Pegboard Time	[28]^b,c,d^
Verbal Fluency	
Controlled Oral Word Association Test (FAS)	[28]^a,b,c,d^
Semantic (Animal) Fluency	[28]^a,b,c,d^

Wechsler Adult Intelligence Scales (WAIS); Paced Auditory Serial Arithmetic Test (PASAT); Normative data corrects for the demographic characteristics indicated by superscript: ^a^Age;^b^ Education; ^c^ Gender; ^d^Ethnicity

The battery was administered and scored by trained psychometricians, supervised by a board-certified neuropsychologist and following standardized procedures, and all measures have excellent reliability and validity [24]. Raw test scores were first converted to T-scores based on the best available demographically corrected normative data [25–30]. Next, T-scores for each individual test were averaged to create mean domain T-scores. Then, all individual test T-scores were averaged to create a mean global T-score [31]. Consistent with prior research, we considered global neurocognitive functioning and neurocognitive domain T-scores < 40 as impaired (i.e., 35–39 mildly impaired, 30–34 mild-moderately impaired, 25–29 moderately impaired, 20–24 moderate-severely impaired, and < 20 severely impaired), T-scores between 40 and 44 as “below average,” and T-scores between 45 and 54 as “average” [31].

#### Depression

We used the Beck Depression Inventory–II (BDI-II) to assess for current depressive symptomatology. The BDI-II is a 21 item self-report scale assessing symptoms of depression over the past 2 weeks [32]. Each of the 21 items contains at least four statements about specific symptoms of depression (e.g., sadness, self-dislike, worthlessness, loss of energy), listed in order of severity from 0 to 3 with a total score ranging from 0 to 63. Severity was defined as: 0–13 for minimal depression, 14–19 for mild depression, 20–28 for moderate depression, and 29–63 for severe depression. We used the computerized Composite International Diagnostic Interview (CIDI) Version 2.1 to assess for lifetime (i.e., current or past) major depressive disorder. The CIDI provides diagnostic information based on Diagnostic and Statistical Manual of Mental Disorders (DSM)-IV criteria [33].

#### Substance use

We also used the CIDI Version 2.1 to assess for lifetime history (i.e., current or past) of substance use disorders [33]. To provide updated DSM-5 nosology about substance use disorders, we defined “use disorder” as meeting criteria for either substance abuse or substance dependence. We used the Addiction Severity Index (ASI; [34]) to assess substance use during the 30 days prior to the baseline evaluation. Specifically, patients were asked how many of the past 30 days they used heroin, other opioids, cannabis, cocaine, amphetamines, alcohol (including use to intoxication), and sedatives, and patients who reported at least one day of use were considered self-reported users. Additionally, urine was collected (unobserved) to assess for use of opioids, cannabinoids, cocaine, amphetamines, and benzodiazepines on the day of the neurocognitive evaluation. These urine tests employed an Enzyme Multiplied Immunoassay Technique (EMIT) analyzed at a commercial laboratory.

### Statistical analyses

Statistical Package for the Social Sciences (SPSS) Version 22.0 was used to analyze all results [35]. Descriptive statistics were calculated to provide information means, standard deviations, percentages, and ranges of relevant demographic data along with neurocognitive, psychiatric, and substance use data. Pearson correlations were used to examine the relationship between current depressive symptomatology and neurocognitive outcomes. A series of independent sample *t*-tests were then computed to examine differences in neurocognitive functioning between participants based on depression and substance use disorder categories (e.g., cannabis, cocaine).

## Results

### Demographic characteristics

Table 2 summarizes the sample’s demographic and clinical characteristics. Almost one-third of the sample was female, and the mean age was 42.2 years (*SD* = 10.3) with a mean of 11.4 years of education (*SD* = 2.3). The majority (52%) was Latinx. The majority of Latinx participants were of Caribbean heritage (Puerto Rican and Dominican). The remainder of the sample was non-Hispanic Black/African American (24%), non-Hispanic white (22%), or of another background (2%).

**Table 2:** Participant demographic and selected clinical characteristics (*N* = 97)

	*M* (*SD*) or % (n)	Range
Demographic characteristics		
Age, years	42.2 (10.3)	21–63
Education, years	11.4 (2.3)	6–18
Female	27% (26)	
HIV +	6% (6)	
Hepatitis C diagnosis^a^	14% (14)	
Race/ethnicity		
Non-Hispanic White	22% (21)	
Non-Hispanic Black/African-American	24% (23)	
Latinx	52% (50)	
Other	2% (3)	
Opioid use characteristics		
SR heroin in last 30 days	87% (79)	
SR other opiates in last 30 days	33% (30)	
UT opiates	98% (92)	
Lifetime opioid use disorder	100% (97)	
Cannabis use characteristics		
SR cannabis in last 30 days	44% (40)	
UT cannabis	25% (24)	
Lifetime cannabis use disorder	44% (42)	
Stimulant use characteristics		
SR cocaine in last 30 days	40% (36)	
UT cocaine	28% (27)	
Lifetime cocaine use disorder	59% (57)	
SR amphetamines in last 30 days	2% (2)	
UT amphetamines	1% (1)	
Sedative use characteristics		
SR alcohol in last 30 days	41% (37)	
SR alcohol to intoxication in last 30 days	24% (22)	
Lifetime alcohol use disorder	54% (52)	
SR sedatives in last 30 days	23% (21)	
UT benzodiazepines	11% (11)	
Lifetime sedative use disorder	28% (27)	
Psychological characteristics		
BDI-II Total Score	15.4 (10.6)	0–45
Minimal total score ≤ 13	50% (45)	
Mild total score 14 and 19	17% (15)	
Moderate total score 20 and 28	23% (22)	
Severe total score 29 and 63	10% (9)	
CIDI lifetime major depressive disorder	37% (35)	

^a^*n* = 85. SR = Self-report use from Addiction Severity Index; UT = Urine toxicology at baseline visit; BDI-II = Beck Depression Inventory – II; CIDI = Composite International Diagnostic Interview–Version 2.1

### Neurocognitive characteristics

Table 3 summarizes neurocognitive characteristics of the sample and prevalence rates of impairment. Estimated premorbid intelligence for the overall sample was below average (*SS* = 87.2, *SD* = 13.2). Consistent with this, global neurocognitive functioning T-score was below average (*M* = 41.6, *SD* = 6.4). Individual neurocognitive domain T-scores ranged from mild-to-moderately impaired for learning (*M* = 34.3, *SD* = 8.3) to average performance in processing speed (*M* = 48.0, *SD* = 8.8). Although all neurocognitive domain T-scores fell within normal limits with the exception of for learning and memory, neurocognitive impairments were still observed in each domain. Over two-thirds of the sample scored in the impaired range for learning (79% impaired) and memory (69% impaired). The remaining prevalence of neurocognitive impairment varied from 18% in speed of information processing, to 42% in motor skills.

**Table 3:** Participant neurocognitive (NC) characteristics based on average T-scores and rates of impairment (*N* = 97)

	*M* (*SD*)	Overall impaired % (*n*)	Mildly impaired % (*n*)	Mild-mod impaired % (*n*)	Moderately impaired % (*n*)	Mod-severely impaired % (*n*)	Severely impaired % *(n*)
WRAT-3 reading (standard score)	87.2 (13.2)	–	–	–	–	–	–
Global NC functioning	41.6 (6.4)	35% (34)	18% (17)	13% (13)	3% (3)	1% (1)	0% (0)
Executive functioning	43.6 (7.7)	35% (34)	24% (23)	9% (9)	2% (2)	0% (0)	0% (0)
Learning	34.3 (8.3)	79% (77)	34% (33)	19% (18)	12% (12)	7% (7)	7% (7)
Memory	35.5 (8.7)	69% (67)	22% (21)	21% (20)	14% (14)	7% (7)	5% (5)
Attention/working memory	43.0 (9.5)	41% (40)	24% (23)	11% (11)	3% (3)	3% (3)	0% (0)
Speed of information processing	48.0 (8.8)	18% (17)	9% (9)	6% (6)	1% (1)	0% (0)	1% (1)
Motor skills	42.0 (10.3)	42% (40)	17% (16)	14% (13)	5% (5)	5% (5)	1% (1)
Verbal fluency	44.5 (9.9)	31% (30)	18% (17)	6% (6)	4% (4)	2% (2)	1% (1)

T-scores between 45 and 54 = average; T-scores between 40 and 44 = below average; T-scores < 40 = overall impaired; T = 35–39 mildly impaired; T = 30–34 mild-moderately impaired; T = 25–29 moderately impaired; T = 20–24 moderate-severely impaired; and T < 20 severely impaired; Mod = moderately

### Psychiatric and substance use comorbidity

Approximately half of the sample endorsed depressive symptomatology, with one-third of the sample endorsing moderate to severe symptoms of depression, and 37% of the sample meeting lifetime criteria for a major depressive disorder.

Lifetime substance use disorder prevalence rates were as follows: 59% cocaine (*n* = 57), 54% alcohol (*n* = 52), 44% cannabis (*n* = 42), and 28% sedatives (*n* = 27). During the 30 days prior to the baseline visit, with the exception of high opioid use (e.g., 87% heroin use) and low amphetamine use (i.e., 2%), self-reported substance use ranged from 23% for sedatives to 44% for cannabis. Urine toxicology results during the baseline visit revealed lower current prevalence rates of these substances ranging from 11% for benzodiazepines to 28% for cocaine (again excluding opiates and amphetamines). Additionally, 50% of the sample tested positive for at least one non-opiate substance (i.e., cannabis, cocaine, amphetamines, benzodiazepines).

### Associations between neurocognitive functioning and depressive symptoms

Current depressive symptomatology was significantly correlated with the global composite of the seven domains (*r* = − 0.23, *p* = 0.03), while the correlations with each of the seven individual domains ranged from *r* = − 0.20, *p* = 0.05 for verbal fluency to *r* = − 0.11, *p* = 0.32 for memory.

Table 4 summarizes differences in the relationship between *lifetime* major depressive disorder and neurocognitive functioning. Participants with a history of lifetime major depressive disorder had significantly worse functioning in two domains: attention/working memory (*t*(94) = 2.72, *p* = 0.01) and motor skills (*t*(92) = 2.64, *p* = 0.01) than those with no lifetime history of major depressive disorder, with medium effect sizes (Cohen’s *d*’s = 0.57–58). Additionally, participants with a history of lifetime major depressive disorder exhibited worse executive functioning, processing speed, and overall global neurocognitive functioning than those without a history of lifetime major depressive disorder at trend levels (*p*’s ≤ 0.10), with modest effect sizes (Cohen’s *d* = 0.35 to 0.40).

**Table 4:** Mean comparisons based on lifetime cannabis use disorder, cocaine use disorder, and major depressive disorder with neurocognitive (NC) functioning (*N* = 97)

Neurocognitive **domain**	Diagnosis of cannabis use disorder				Diagnosis of cocaine use disorder				Diagnosis of major depressive disorder			
	Yes (*n *= 42)	No (*n *= 54)			Yes (*n *= 57)	No (*n *= 39)			Yes (*n *= 35)	No (*n *= 61)		
	*M *(*SD*)	*M *(*SD*)	*t*-test	Cohen’s *d*	*M *(*SD*)	*M *(*SD*)	*t*-test	Cohen’s *d*	*M *(*SD*)	*M *(*SD*)	*t*-test	Cohen’s *d*
Global NC functioning	42.7 (5.7)	40.7 (6.9)	− 1.5	0.31	42.1 (6.7)	40.8 (6.1)	− 1.0	0.20	39.9 (7.0)	42.5 (6.0)	1.9*	0.41
Executive functioning	45.6 (7.3)	42.1 (7.9)	− 2.3**	0.46	44.9 (8.1)	41.7 (6.9)	− 2.0*	0.42	41.6 (8.0)	44.7 (7.5)	1.9*	0.40
Learning	35.3 (9.1)	33.6 (7.3)	− 1.0	0.21	33.6 (7.8)	35.4 (9.0)	1.0	0.22	33.2 (7.7)	35.0 (8.6)	1.0	0.22
Memory	36.4 (8.1)	34.7 (9.3)	− 0.9	0.19	35.1 (8.5)	36.1 (9.3)	0.5	0.11	34.7 (8.7)	35.9 (8.9)	0.6	0.14
Attention/working memory	46.0 (9.9)	40.8 (8.6)	− 2.8***	0.57	45.3 (9.6)	39.8 (8.5)	− 2.9***	0.60	39.7 (9.2)	45.0 (9.2)	2.7***	0.58
Speed of information processing	47.9 (7.4)	48.2 (10.0)	0.1	0.03	48.7 (9.3)	47.1 (8.3)	− 0.9	0.18	46.0 (9.6)	49.2 (8.3)	1.7*	0.36
Motor skills	42.1 (9.5)	41.9 (11.0)	− 0.1	0.02	41.2 (10.5)	43.2 (10.1)	0.9	0.19	38.4 (9.4)	44.1 (10.3)	2.6**	0.57
Verbal fluency	45.7 (9.1)	43.8 (10.5)	− 1.5	0.19	46.0 (10.7)	42.6 (8.3)	− 1.7*	0.35	46.0 (10.9)	43.9 (9.3)	− 1.0	0.21

Mean and Standard Deviations (*M*, *SD*) based on average T scores; Diagnosis assessed using the Composite Diagnostic Interview (CIDI); T-scores between 45 and 54 = average; T-scores between 40 and 44 = below average; T-scores < 40 = overall impaired; T = 35–39 mildly impaired; T = 30–34 mild-moderately impaired; T = 25–29 moderately impaired; T = 20–24 moderate-severely impaired; and T < 20 severely impaired

^*^p < 0.10

^**^p < 0.05

^***^p < 0.01

### Associations between neurocognitive functioning and substance use disorders

Table 4 also summarizes differences in the relationship between lifetime substance use disorder categories, global neurocognitive functioning, and each of the seven neurocognitive domains. Participants with a lifetime history of cannabis use disorder had better executive functioning *t*(94) =  − 2.25, *p* = 0.03) than those without a lifetime history of cannabis use disorder with a medium effect size (Cohen’s *d* = 0.46). Similarly, participants with cannabis use disorder also performed better on attention/working memory tasks *t*(94) =  − 2.77, *p* = 0.007) with a medium effect size (Cohen’s *d* = 0.56). There were no other differences between participants with and without a history of cannabis use disorder globally or in any other neurocognitive domain.

Participants with a lifetime history of cocaine use disorder outperformed those with no lifetime history of cocaine use disorder on attention/working memory tasks *t*(94) =  − 2.90, *p* = 0.005) with a medium effect size (Cohen’s *d* = 0.61). Additionally, participants with a lifetime history of cocaine use disorder had better executive functioning *t*(94) = -2.00, *p* = 0.06) and verbal fluency *t*(94) =  − 1.69, *p* = 0.10) at trend levels with medium effect sizes (Cohen’s *d* = 0.36 to 0.41). There were also no differences between participants with and without a history of cocaine use disorder globally or in any other neurocognitive domain.

Participants with or without a lifetime history of alcohol use disorder or sedative use disorder also did not differ on any neurocognitive domain.

## Discussion

We found that the vast majority of our diverse sample of OUD patients who were initiating OAT with methadone or buprenorphine/naloxone were impaired in domains of learning and memory. Major depressive disorder was also common, and half the sample reported current depressive symptomatology. Lifetime prevalence of substance use disorders were high with over half of the sample reporting a lifetime history of alcohol or cocaine use disorder, 44% reporting lifetime cannabis use disorder, and 28% reporting lifetime sedative use disorder. As expected, current depressive symptoms and a lifetime history of major depressive disorder were both negatively related to specific domains of neurocognitive functioning (e.g., attention/working memory, motor skills). However, contrary to our hypothesis, we did not find a negative relationship between lifetime diagnoses of alcohol or cocaine use disorder and neurocognitive functioning. Instead, we found no differences in neurocognitive functioning between those with and without a history of alcohol use disorder. Moreover, we found that attention/working memory was significantly *better* in individuals with a history of cocaine or cannabis use disorder, and executive functioning was significantly better in individuals with a history of cannabis use disorder.

We found a similar prevalence of learning and memory impairment and impairment in other neurocognitive domains as previous studies [5–9, 36]. For instance, compared to Arias and colleagues [5] we found the following impairment rates: learning: 79% present study vs. 73% Arias and colleagues [5], memory: 69% present study vs. 68% Arias and colleagues [5], attention/working memory: 41% present study vs. 36% Arias and colleagues [5], and executive functioning 35% present study vs. 44% Arias and colleagues [5]. The especially high prevalence of learning and memory impairment in persons with OUD who are initiating OAT is not surprising given that chronic opioid use decreases temporal lobe gray matter density and results in corresponding decreases in hippocampal neurogenesis [37, 38]. Additionally, unlike other neurocognitive domains, learning and memory impairment appears to be independent of common comorbidities (i.e., depression and substance use disorders) in this sample.

Similar to previous studies, we found that depression was common in OUD patients [5, 17, 22]. Similar to Loeber and colleagues [22], our study found that depression (i.e., lifetime major depressive disorder and current depressive symptomatology) negatively impacted neurocognitive performance (i.e., globally and domains of attention/working memory and motor skills), but other studies have not found a relationship between current or past depression and cognitive functioning [5, 12] in OUD patients. In contrast to our study, Sanborn and colleagues [12] investigated this relationship in patients already taking methadone (rather than in patients starting OAT). Arias and colleagues [5] studied a small sample and may not have had sufficient power to detect an impact of depression on cognitive functioning, though they did report a negative but non-significant relationship between motor skills and lifetime history of major depressive disorder with a similar effect size (i.e., Cohen’s *d* = 0.70) as we found. Because we found a significant negative relationship for both current and lifetime depression and neurocognitive outcomes in persons with OUD who are starting OAT, our findings have increased generalizability compared to past studies. However, given the simple bivariate relationship between depression and cognition in the present study, future research should clarify the specific role of current and lifetime history of depression in negatively impacting neurocognitive performance in OUD patients.

In contrast to Arias and colleagues [5], results of the present study revealed that cocaine and cannabis use disorder diagnoses were *positively* associated with neurocognitive functioning in specific domains. It is possible that the difference in diagnostic criteria (DSM-IV in Arias vs. DSM-5 in present study), along with the increased power via larger sample size, might partially explain this difference in findings. The positive relationship between attention/working memory and cocaine use is somewhat consistent with previous research [39]. Specifically, Byrd and colleagues [39] found a positive association between positive urine toxicology results for cocaine and attention/working memory in a sample of HIV ± polysubstance users, including current opioid users. These converging findings may be due to the “neuro-activating” properties of cocaine that could result in enhanced attentional performance. Future research should explore whether potential neuro-activating properties of cocaine in polysubstance users persists overtime as in those with a lifetime history of cocaine use disorder. The current study’s finding regarding the positive association between lifetime cannabis use disorder and cognitive functioning is both consistent [40] and inconsistent [41] with limited prior literature. For instance, Gruber and colleagues [40] reported improved executive functioning performance over time in medical marijuana users. Ultimately, our results appear to indicate that prior cocaine or cannabis use disorder do not confer a risk for poor neurocognitive functioning in patients entering OAT.

This study has several important implications and strengths. First, by characterizing the prevalence of neurocognitive impairment, depression, and other substance use in persons with OUD who are starting OAT, our results provide useful information to treatment providers. This knowledge might allow clinicians to help patients taking OAT develop compensatory strategies for poor neurocognitive functioning (e.g., learning and memory), while providing adjunctive treatment for depression. Second, our study was able to successfully replicate the prevalence of neurocognitive impairment, depression, and comorbid substance use reported in our previous study by Arias and colleagues [5]. We also were able to provide the additional power necessary to detect a statistically significant association between depression and neurocognitive functioning, while uncovering that OAT-initiating persons with a lifetime history of comorbid substance use disorders did not have greater neurocognitive impairments than those without. Although our findings must still be replicated in different patient populations and in different locations, we are confident in the validity of these results. Third, we used recent and well-validated measures of neurocognitive functioning, depression, and substance use, which addresses important limitations of some prior studies on this topic (e.g., [5, 42, 43]). Fourth and finally, the high racial/ethnic diversity of the sample is both a strength and a limitation. The present study fills an important gap in the currently sparse literature on characteristics of racial/ethnic minority patients entering OAT. However, our predominantly Latinx sample of Caribbean heritage (Puerto Rican and Dominican) may not generalize to persons from other racial/ethnic groups and/or regions of the U.S. [44, 45]. Future studies should replicate these findings in other racial/ethnic groups (e.g., non-Hispanic white, Asian American, American Indian/Alaska Native) and settings (e.g., rural, suburban).

Despite these strengths, our study has several limitations. The cross-sectional nature of our research design limits the generalizability of our results. Specifically, these findings only apply to patients early in OAT (i.e., within the first 14 days) and do not account for individuals who will drop out of treatment. Therefore, it remains to be seen if these high prevalence rates of neurocognitive impairment, depression, and other substance use are present in patients who remain in treatment long-term. Future longitudinal studies should be conducted to provide characteristics of OAT patients over time. Finally, because we had no healthy control group, it is unknown if our findings are applicable to OUD patients in general or just those seeking OAT. Future studies might incorporate a non-treated control group to determine if these findings are directly related to initiating OAT.

## Conclusions

We found high prevalence rates of neurocognitive impairment, depression, and other substance use among diverse persons with OUD who were starting OAT. The vast majority of these patients exhibited impairments in learning and memory, and current depression and substance use, especially cannabis, cocaine, and alcohol, were common. Depressed patients were especially likely to have neurocognitive impairments, but patients with a lifetime history of either cannabis or cocaine use disorder had no worse neurocognitive functioning than those without. Treatment providers should be aware that patients starting OAT may present with neurocognitive and psychiatric complications, and depression in these patients might be associated with especially poor neurocognitive outcomes. This knowledge might allow clinicians to help patients taking OAT develop compensatory strategies for poor neurocognitive functioning (e.g., learning and memory), while providing adjunctive treatment for depression.

## Data Availability

The datasets used and/or analyzed during the current study are available from the corresponding author on reasonable request.

## Abbreviations

OAT: Opioid agonist treatment
OUD: Opioid use disorder
OTP: Opioid treatment program
U.S.: United States
DoSA: Division of substance abuse
WRAT-3: Wide Range Achievement Test-Reading subtest, 3rd edition
BDI-II: Beck depression inventory–II
CIDI: Composite International Diagnostic Interview
DSM: Diagnostic and Statistical Manual of Mental Disorders
ASI: Addiction Severity Index
EMIT: Enzyme Multiplied Immunoassay Technique
SPSS: Statistical Package for the Social Sciences
